# MetageneCluster: a Python package for filtering conflicting signal trends in metagene plots

**DOI:** 10.1186/s12859-024-05647-3

**Published:** 2024-01-12

**Authors:** Clayton Carter, Aaron Saporito, Stephen M. Douglass

**Affiliations:** Connecticut College, New London, CT USA

**Keywords:** Metagene plot, Sequencing data, k-means clustering, Visualization

## Abstract

**Background:**

Metagene plots provide a visualization of biological signal trends over subsections of the genome and are used to perform high-level analysis of experimental data by aggregating genome-level data to create an average profile. The generation of metagene plots is useful for summarizing the results of many sequencing-based applications. Despite their prevalence and utility, the standard metagene plot is blind to conflicting signals within data. If multiple distinct trends occur, they can interact destructively, creating a plot that does not accurately represent any of the underlying trends.

**Results:**

We present MetageneCluster, a Python tool to generate a collection of representative metagene plots based on k-means clustering of genomic regions of interest. Clustering the data by similarity allows us to identify patterns within the features of interest. We are then able to summarize each pattern present in the data, rather than averaging across the entire feature space. We show that our method performs well when used to identify conflicting signals in real-world genome-level data.

**Conclusions:**

Overall, MetageneCluster is a user-friendly tool for the creation of metagene plots that capture distinct patterns in underlying sequence data.

**Supplementary Information:**

The online version contains supplementary material available at 10.1186/s12859-024-05647-3.

## Background

The development and rapid growth of high-throughput sequencing technologies has led to the need for ways to visualize large-scale biological experiments. A common way to summarize sequencing data is to aggregate and average signal coverages over many regions to generate a single graph that summarizes the collective regions, termed a metagene plot. Metagene plots are used to analyze data derived from a variety of biological experiments, including bisulfite (BS) sequencing [1], chromatin immunoprecipitation (ChIP) sequencing [2], RNA sequencing [3], and ribosome profiling [4]. Many pipelines now exist for quickly and efficiently generating these graphs, including method-specific [5–7] and generalized [8, 9] software packages. These plots are useful for distilling many individual signals, often tens of thousands of genes, into a single graphic. Despite their utility, existing methods are limited in that they produce only a single plot that represents all constituent genomic loci, potentially losing valuable information in the process. For instance, consider a situation in which one pattern has a sharp spike at the start of the gene while another a sharp spike at the end. The resultant plot may more closely resemble a plateau across the entire gene depicting levels somewhere between the peak and trough of each of the constituent trends. Our method is the first to seek to separate genomic loci to generate more than a single metagene plot from a single alignment file when conflicting trends exist.

Clustering methods are unsupervised data mining techniques that seek to find structure within data [10, 11]. These methods have been used in bioinformatics to find patterns in gene expression data [12], reconstruct phylogenetic relationships [13], and predict gene features [14, 15]. One such method of clustering is k-means clustering. K-means uses a fixed number of clusters, initializing each cluster’s center randomly and assigning each data point to the nearest cluster center. The clusters then move to the center of the data points that they represent. This process continues until a desired condition is met, such as convergence.

The MetageneCluster software package allows researchers to produce metagene plots from sequence data that have been grouped by k-means clustering. Our implementation allows users to process high-throughput sequence data directly from sam format alignment and gtf/gff3 annotation files. We validate our method on RNA-seq and ChIP-seq data taken from three different organisms.

## Implementation

**Fig. 1:**
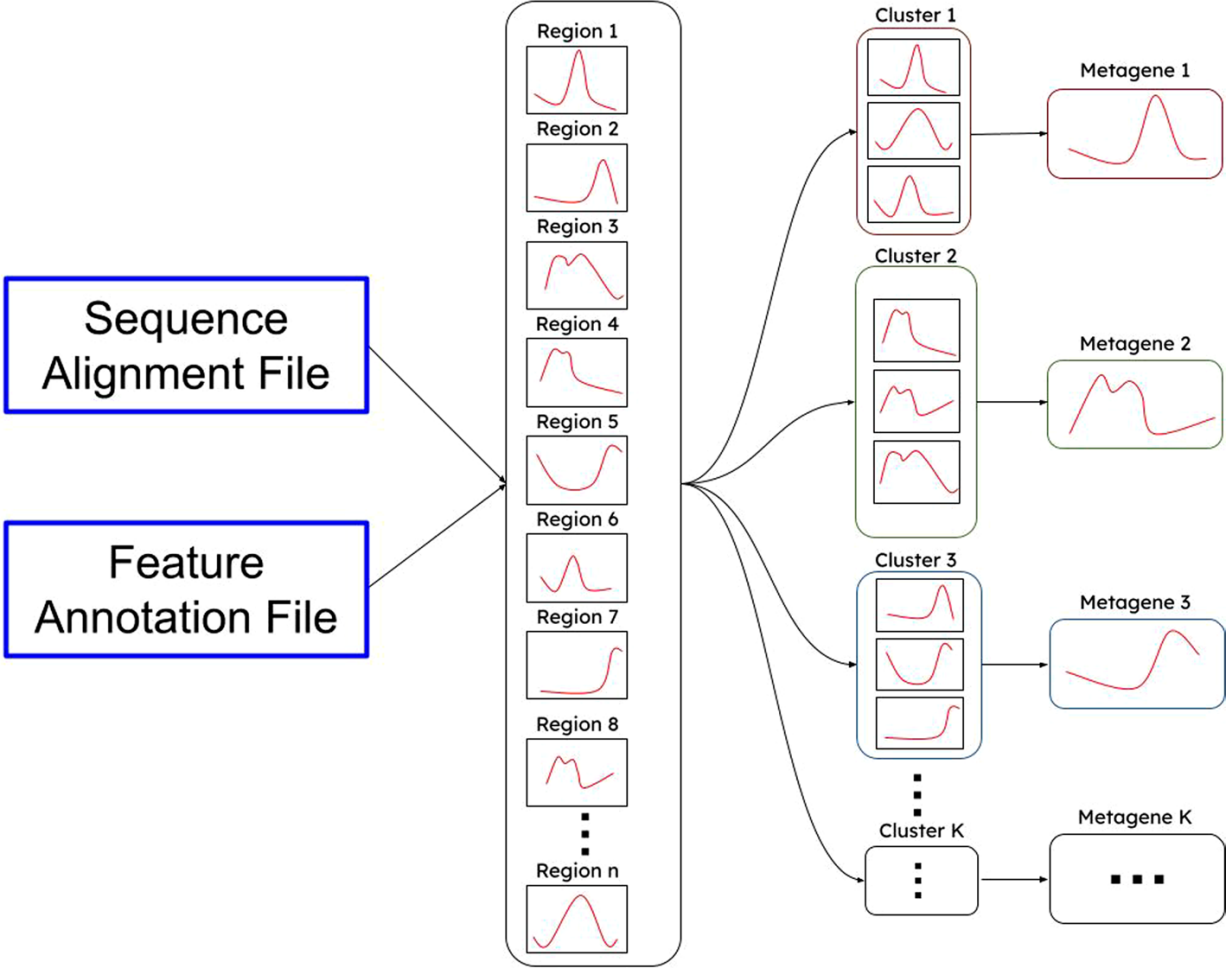
Flowchart demonstrating the functionality of MetageneCluster. The user provides an annotation file, an alignment file, and genomic features to be analyzed. Each feature has its coordinates found in the annotation file, has its coverage determined from the alignment file, and then has its coverage normalized by length. K-means clustering then groups each feature into its most representative metagene plot and exports each plot and the corresponding list of features

The aim of MetageneCluster is to provide users with metagene plots that better reflect the variability within large-scale biological sequence data. To achieve this, MetageneCluster works in several steps. First, user input is collected, and the annotation and alignment files are read in. Then, coverage across each feature of interest is calculated and normalized according to length. K-means clustering is then performed to group features with similar patterns. Finally, each metagene plot is generated using MatPlotLib, and the list of features that constitute each plot is printed in a text file. A graphical summary of the major steps in this workflow is shown in Fig. 1.

### Length normalization of features

The start and end coordinates of features are extracted from the annotation file provided by the user, and the alignment files are read to calculate the coverage across each feature. In many applications, length will vary among instances of a particular feature type. Therefore, the length of each feature must be normalized to a standard before they can be compared. Furthermore, this must be done in a way in which the overall shape of each coverage graph is preserved. To achieve this, we calculate the normalization ratio as the ratio of each feature’s length to the standard we are normalizing to. The normalized vector results from averaging the initial vector over windows of size equal to the normalization ratio.

### K-means clustering of feature graphs

After length-normalization, vectors built from each feature are clustered by the k-means algorithm. For any given number of clusters, k, MetageneCluster begins by generating k plots with values at each normalized point along the feature length selected at random within the range of values present in the data. The distance between every feature and each cluster is calculated by summing the distance between the feature and the cluster at each point along the x-axis. Each feature is then grouped into the cluster to which it is closest to. Each of the k plots are then recalculated to be the average at each position along the x-axis of the features that are assigned to it. The distances to the new clusters are calculated again, features are reassigned, and average clusters are recalculated. This process continues until convergence is achieved. MetageneCluster utilizes this procedure for increasing values of k, starting at k = 1, until the total distance between all features and their representative centers fails to decrease by more than a user-specified parameter with a default value of 20%.

### Workflow integration

User input is required in sam format for alignment files and gff format for annotation files. Therefore, MetageneCluster can work downstream of any alignment software that produces sam formatted files or a filetype that can be converted to sam format. In addition to the plots themselves, the method outputs the names of all loci that comprise each cluster in text files, allowing for further downstream analyses, such GO [16].

### Dependencies

MetageneCluster is written for, validated, and tested using Python3 (version 3.9.16). The method requires numpy for mathematical computation and matplotlib for visualization of plots [17, 18].

### Efficiency

From a runtime perspective, the method works in three main steps: reading in the alignment and annotation data, identifying the regions of interest from the annotation data and building an array for each of these loci of interest, and then the iterative clustering itself. Reading in the data scales linearly with respect to the total number of alignment results and the total number of features in the annotation file. From the alignment data, we produce one coverage array per chromosome, making the total number of aligned reads unimportant for the runtime from this point onward. Once the regions of interest are identified, we build one array per locus and the clustering algorithm works on this set of arrays. The creation of these arrays scales linearly with the number of loci of interest, here termed “n.” For the clustering algorithm, we compute the distance between each of these n loci and the iteratively improving cluster centers, k. Each step of this process scales linearly with respect to n times the size of the arrays, as determined by the user, times the number of cluster centers, k. While each step is relatively fast and scales well, the number of steps that must occur to reach convergence will differ depending on the final value of k as well as how far the initial randomly selected cluster centers are from the centers in the solution. Importantly, the step with the most potential variation in computation scales only with the number and size of the features of interest, not the number of alignments, so overall performance should remain similar as improving technology increases the number of reads that are produced by sequencing experiments.

In terms of what this runtime translates to practically, we tested on a machine with a 14 core, 2.6 GHz processor. For a human dataset with approximately 23 million lines in the alignment file, approximately 3 million lines in the annotation file, and 484,033 loci of interest, the method took roughly 11 min to read in the data, 4 min to generate the arrays to cluster, and 52 min to compete the k-means clustering, for a total runtime of roughly 67 min. For a yeast dataset with approximately 81 million lines in the alignment file, approximately 23,000 lines in the annotation file, and 6897 loci of interest, the method took roughly 10 min to read in the data, 10 s to generate the arrays to cluster, and 30 s to complete the k-means clustering, for a total runtime of roughly 11 min.

## Results

In this section, we validate the utility of our method on three datasets. We use a diverse range of organisms and data sources for the most broadly representative demonstration of effectiveness. Resultant clustered metagene plots and their constituent genes are analyzed to find differences between clusters and to compare their shape with a single, unclustered plot.

**Fig. 2:**
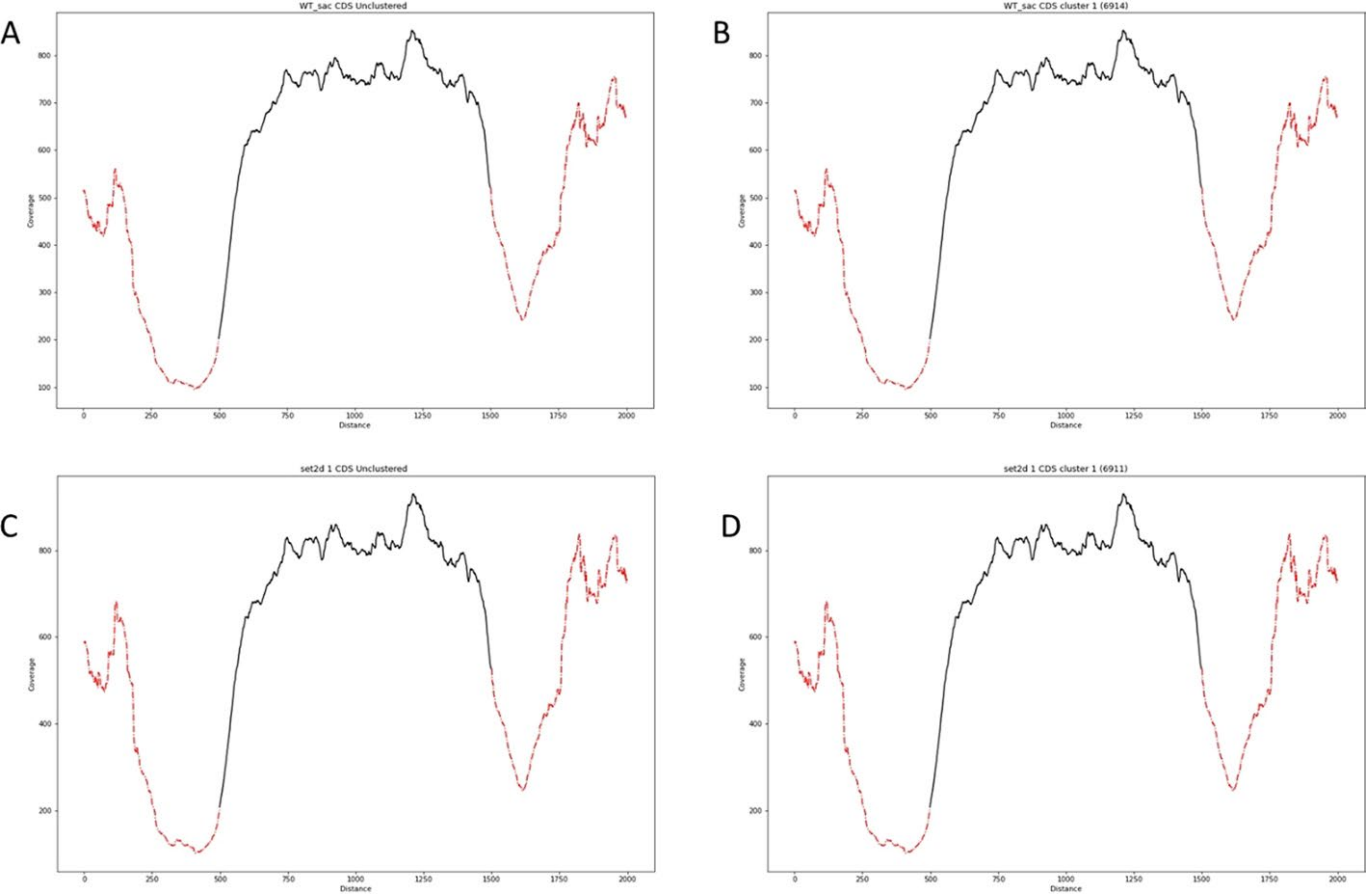
Performance of MetageneCluster on CDS loci from *Saccharomyces cerevisiae* RNA-sequence data. MetageneCluster produces a single cluster for wildtype strain (**B**) and for *set2Δ* strain (**D**) under default parameters. These graphs look virtually identical to the unclustered wildtype (**A**) and *set2Δ* (**C**) plots

The first dataset is derived from an RNA sequence experiment performed in *Saccharomyces cerevisiae* that analyzes the effect of mutant strains on proper spliceosome assembly [19]. We use the wildtype and *set2* deletion (*set2Δ*) strains and cluster based on all coding sequences (CDS). Figure 2 shows the results for each strain. For this dataset, we find that all data clusters in a single cluster under default conditions in both wildtype and *set2Δ*. This suggests that the pattern of gene expression over the course of CDS sequences does not vary substantially among genes in either strain.

**Fig. 3:**
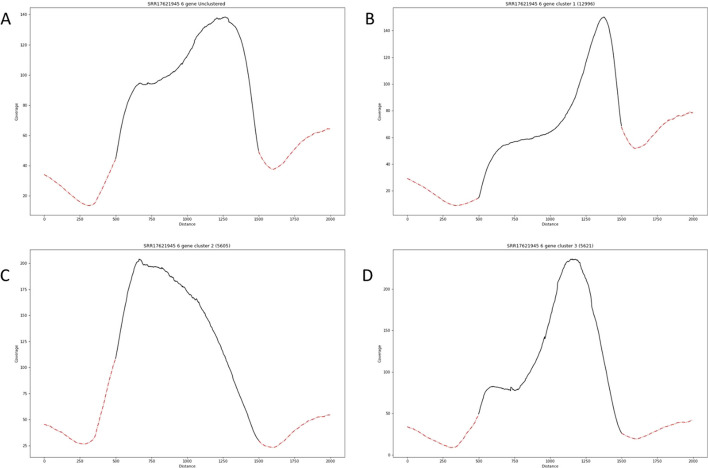
Performance of MetageneCluster on gene loci from *Arabidopsis Thaliana* RNA-sequence data. Comparison of unclustered (**A**) and clustered (**B**–**D**) plots generated from *met1-1* strain RNA sequencing

We also analyze RNA sequence data generated in *Arabidopsis Thaliana.* We select the *met1-1* strain data taken from a study investigating heterochromatin marks influencing DNA methylation profiles [20]. Using this dataset, our method creates three distinct clustered patterns (Fig. 3). We utilize Gene Ontology (GO) analysis to find biologically relevant differences between the genes that compose these three clusters [16, 21, 22]. We focus on cluster 2 (Fig. 3C) because it has the greatest dissimilarity in shape when compared to the unclustered metagene plot. Cluster 2 is de-enriched for genes involved in negative regulation of gene expression as well as epigenetic regulation of gene expression and enriched in genes involved in cellular response to hypoxia, auxin, cold, water deprivation, and other stimuli (Additional file 1: Table S1). Our results suggest that the *met1-1* mutant strain leads to a difference in the expression pattern of genes involved in hormonal response and gene expression.

**Fig. 4:**
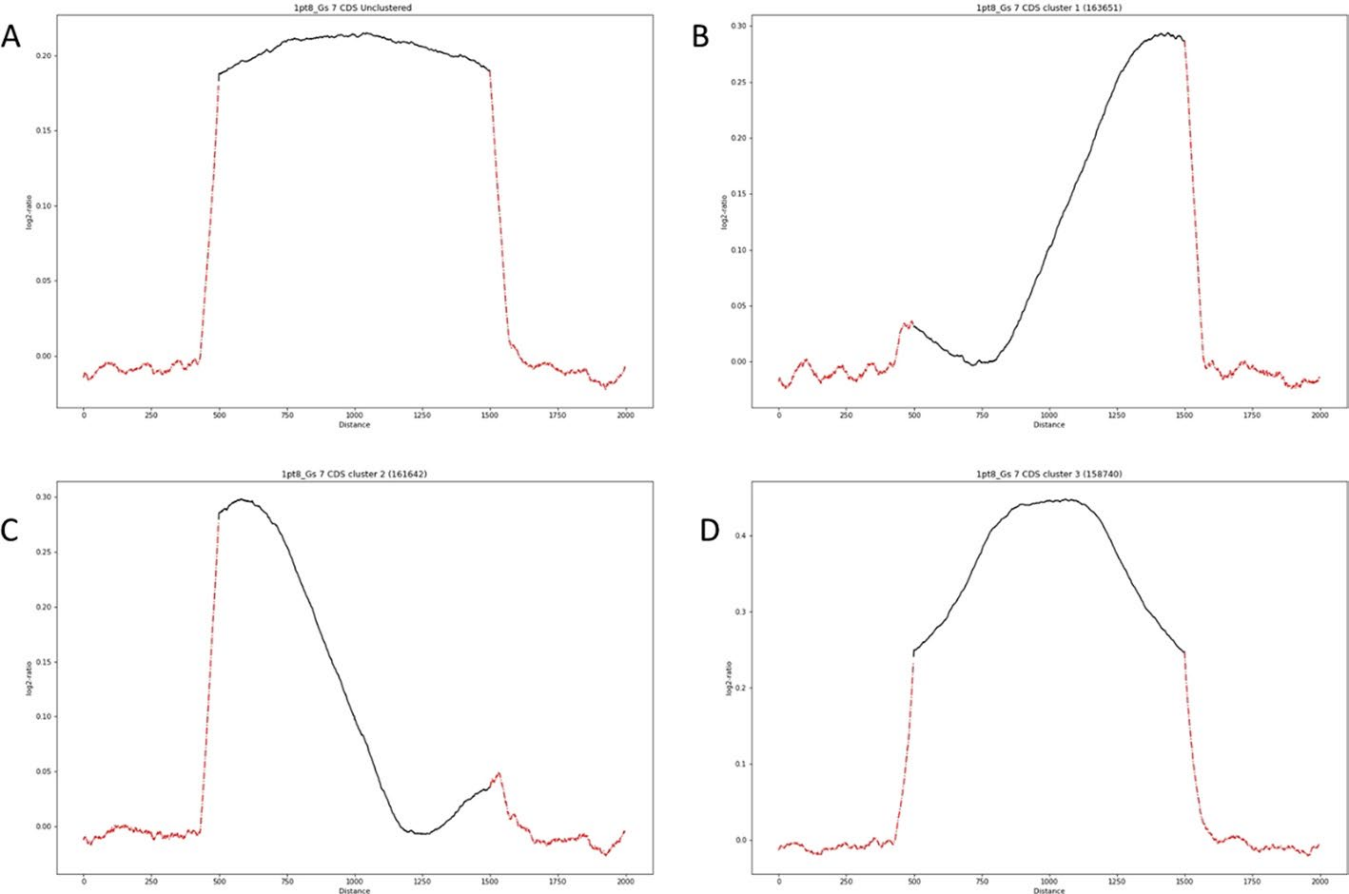
Performance of MetageneCluster on CDS loci from *Homo sapiens* ChIP-sequence data. Comparison unclustered (**A**) and clustered (**B**–**D**) plots generated from log2-ratio of polymerase II binding in macrophages exposed to 1.8× gravity versus control

Our final test set is *Homo sapiens* ChIP-seq data. We apply MetageneCluster to samples of in vitro macrophage cells exposed to 1.8× gravitational force as compared to standard, 1× gravity with an RNA polymerase II chip antibody [23]. Our method finds three distinct cluster profiles in CDS regions (Fig. 4). While all clusters show slightly greater RNA polymerase II binding across CDS regions in high gravity, one cluster has the greatest increase in occupancy at the 5′ end, one at the 3′ end, and one in the center. This is in contrast to the unclustered plot, which makes it appear that there is a relatively flat increase in occupancy across all genes.

Together, our results indicate that next-generation sequence data signals are often composed of conflicting trends and that averaging across all regions of interest can cause these trends to be combined destructively.

## Conclusions and future directions

In this paper, we developed MetageneCluster, a Python package that employs k-means clustering to group otherwise conflicting signal trends in metagene plots. By clustering, we allow users to construct multiple metagene plots from a single alignment file. Each resultant plot is composed of genomic regions that show similar patterns among the constituent loci. We tested our method on three different sequencing applications using a model plant, animal, and fungus. Our results demonstrate that the metagene plots built from these clusters are able to discover important biological nuance across a variety of sequencing applications and organisms. Presently, MetageneCluster produces clusters of genomic loci using only k-means clustering. K-means is a time-efficient methodology for performing clustering, but it has few algorithmic guarantees due to the initial random placement of clusters. In the future, we plan to add support for the user to cluster loci using hierarchical clustering to compensate for these limitations at the cost of likely substantially increased runtime. As access to increased computational power improves, we anticipate this tradeoff may become more attractive to potential users.

## Availability and requirements

- Project Name: MetageneCluster
- Project home page: https://github.com/aasaporito/MetageneCluster
- Operating System(s): Platform independent
- Programming language: Python
- Other requirements: matplotlib, numpy
- License: Mozilla Public License, v. 2.0
- Restrictions to use by non-academics: None

### Supplementary Information

**Additional file 1: Table S1.** GO biological process enrichment analysis of Arabidopsis thaliana met1-1 RNA sequence data.

## Data Availability

The datasets analyzed during the current study are available in the National Library of Medicine Gene Expression Omnibus repository, available at https://www.ncbi.nlm.nih.gov/geo/. Accession numbers are SRR7852780 (*Saccharomyces cerevisiae* wildtype RNA-seq), SRR7852782 (*Saccharomyces cerevisiae* set2Δ RNA-seq), SRR17621945 (*Arabidopsis thaliana* met1-1 RNA-seq), SRR22827387 (*Homo sapiens* 1.8× gravity ChIP-seq), and SRR22827393 (*Homo sapiens* 1× gravity ChIP-seq).

## Abbreviations

BS-seq: Bisulfite sequencing
CDS: Coding sequence
ChIP-Seq: Chromatin immunoprecipitation sequencing
GFF3: General Feature Format 3
GO: Gene Ontology
GTF: General transfer format
RNA: Ribonucleic acid
SAM format: Sequence Alignment Map format
